# 3D printing of injury-preconditioned secretome/collagen/heparan sulfate scaffolds for neurological recovery after traumatic brain injury in rats

**DOI:** 10.1186/s13287-022-03208-0

**Published:** 2022-12-19

**Authors:** Xiao-Yin Liu, Zhe-Han Chang, Chong Chen, Jun Liang, Jian-Xin Shi, Xiu Fan, Qi Shao, Wei-Wei Meng, Jing-Jing Wang, Xiao-Hong Li

**Affiliations:** 1 Academy of Medical Engineering and Translational Medicine, Tianjin University, Tianjin, 300072 China; 2 Department of Neurosurgery, West China Hospital, West China Medical School, Sichuan University, Chengdu, 610041 Sichuan China; 3 Tianjin Key Laboratory of Neurotrauma Repair, Characteristic Medical Center of People’s Armed Police Forces, Tianjin, 300162 China

**Keywords:** Traumatic brain injury, 3D printing, Biomaterial scaffolds, Injury-preconditioned secretome, Human umbilical cord blood mesenchymal stem cells, Neural reconstruction

## Abstract

**Background:**

The effects of traumatic brain injury (TBI) can include physical disability and even death. The development of effective therapies to promote neurological recovery is still a challenging problem. 3D-printed biomaterials are considered to have a promising future in TBI repair. The injury-preconditioned secretome derived from human umbilical cord blood mesenchymal stem cells showed better stability in neurological recovery after TBI. Therefore, it is reasonable to assume that a biological scaffold loaded with an injury-preconditioned secretome could facilitate neural network reconstruction after TBI.

**Methods:**

In this study, we fabricated injury-preconditioned secretome/collagen/heparan sulfate scaffolds by 3D printing. The scaffold structure and porosity were examined by scanning electron microscopy and HE staining. The cytocompatibility of the scaffolds was characterized by MTT analysis, HE staining and electron microscopy. The modified Neurological Severity Score (mNSS), Morris water maze (MWM), and motor evoked potential (MEP) were used to examine the recovery of cognitive and locomotor function after TBI in rats. HE staining, silver staining, Nissl staining, immunofluorescence, and transmission electron microscopy were used to detect the reconstruction of neural structures and pathophysiological processes. The biocompatibility of the scaffolds in vivo was characterized by tolerance exposure and liver/kidney function assays.

**Results:**

The excellent mechanical and porosity characteristics of the composite scaffold allowed it to efficiently regulate the secretome release rate. MTT and cell adhesion assays demonstrated that the scaffold loaded with the injury-preconditioned secretome (3D-CH-IB-ST) had better cytocompatibility than that loaded with the normal secretome (3D-CH-ST). In the rat TBI model, cognitive and locomotor function including mNSS, MWM, and MEP clearly improved when the scaffold was transplanted into the damage site. There is a significant improvement in nerve tissue at the site of lesion. More abundant endogenous neurons with nerve fibers, synaptic structures, and myelin sheaths were observed in the 3D-CH-IB-ST group. Furthermore, the apoptotic response and neuroinflammation were significantly reduced and functional vessels were observed at the injury site. Good exposure tolerance in vivo demonstrated favorable biocompatibility of the scaffold.

**Conclusions:**

Our results demonstrated that injury-preconditioned secretome/collagen/heparan sulfate scaffolds fabricated by 3D printing promoted neurological recovery after TBI by reconstructing neural networks, suggesting that the implantation of the scaffolds could be a novel way to alleviate brain damage following TBI.

**Graphical abstract:**

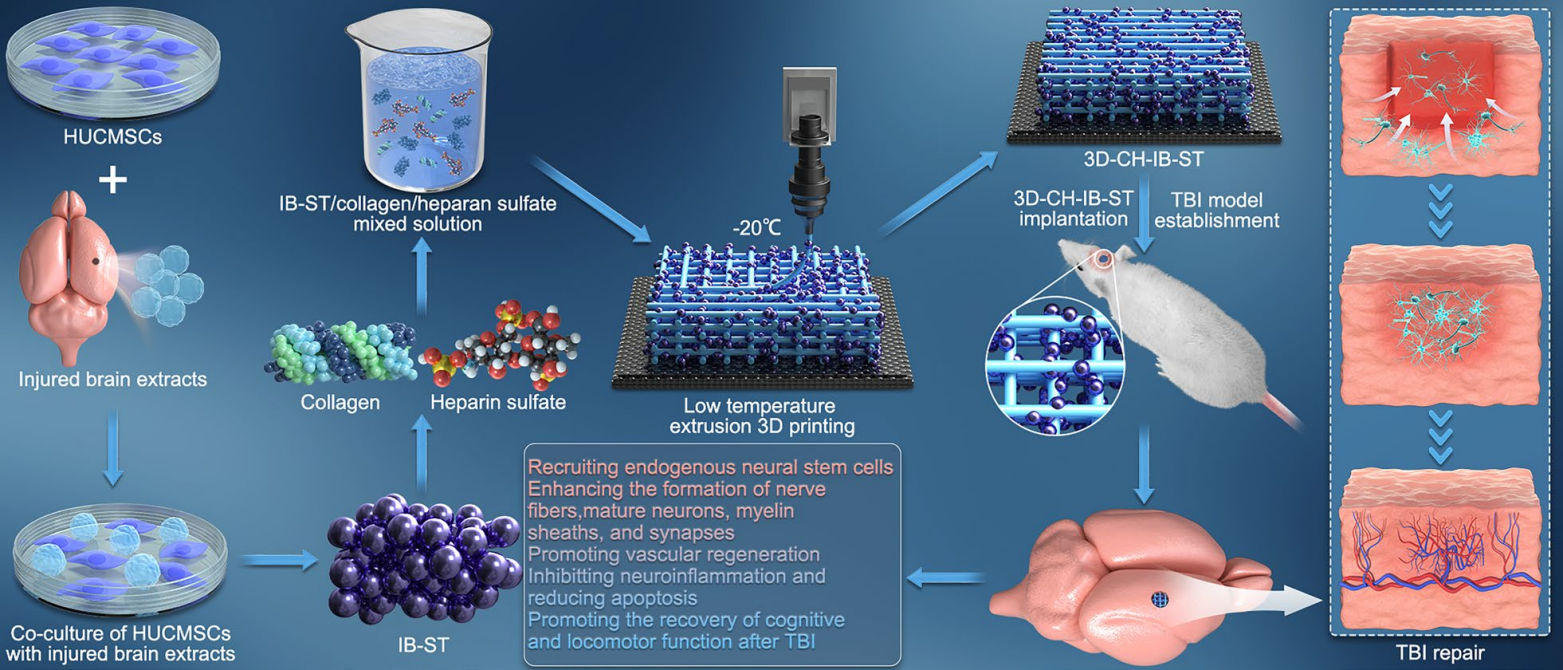

**Supplementary Information:**

The online version contains supplementary material available at 10.1186/s13287-022-03208-0.

## Introduction

Traumatic brain injury (TBI) is a major global health problem that often leads to physical disability or death, but there is still no effective clinical treatment [1, 2]. Classic methods for traumatic brain injury, including rescue surgery, routine drugs, and functional exercise, have already made significant progress; however, further secondary injury responses, including axonal injury, glial scarring, neuroinflammation, and accumulation of harmful substances in the lesion microenvironment, have been identified [3, 4]. Together, these factors form a barrier that prevents structural and functional recovery. To enhance exogenous delivery to the lesion area after TBI, biomaterial scaffolds combined with various factors, drugs, and stem cells have been tested as a therapeutic strategy [5–12]. Unfortunately, developing materials that can break the above barriers is still a challenge.

The choice of biomaterials has been explored for many years. Due to its biocompatibility, collagen is widely acknowledged as a viable biomaterial scaffold in tissue engineering as a natural polymer [13–18]. However, collagen's ability to support may be hampered by its weak mechanical and thermal qualities. Due to the faster degradation rate, it also limits its role in TBI therapy [19, 20]. Heparan sulfate is a glycosaminoglycan family linear polysaccharide that is generated by nearly all animal cells and occurs on the cell surface. It has been confirmed that heparin is cross-linked with extracellular proteins and improves the strength of the scaffold [21]. We employed heparan sulfate as a cross-linker in order to take advantage of collagen’s benefits while avoiding its disadvantages. Along with enhanced mechanical qualities and thermal stability, heparan sulfate also binds growth factors in large amounts, protecting them from protease degradation and enhancing and prolonging the activity of growth factors [22]. A previous study confirmed that collagen/heparan sulfate scaffolds promote recovery of locomotor function after TBI [23].

Human umbilical cord blood mesenchymal stem cells (HUCMSCs) are widely used for functional cell replacement after central nervous system (CNS) disease because of their immunomodulatory and self-renewal capacity [24–27]. However, their efficacy may be limited due to the death of transplanted cells and the inflammatory response of the surrounding tissue. Recent studies have shown that transplanted MSCs exert therapeutic effects through paracrine mechanisms and that the secretome plays an important role in this process [17, 18, 28–33]. The secretome of MSCs contains many growth factors and cytokines, including TGFB1, FGF2, and BDNF [34, 35]. Since the secretome component is dependent on the local microenvironment, preconditioning MSCs leads to alteration of their contents [36]. A study confirmed that the secretome of MSCs preconditioned with hypoxia enhanced the therapeutic effect on TBI [37]. Microvesicles derived from brain-extract-treated mesenchymal stem cells have been shown to enhance neurological functioning in a rat model of ischemic stroke [38]. Our previous study demonstrated that injury-preconditioned secretome of HUCMSCs enhanced cognitive function recovery in rats after TBI [39]. It is widely accepted that preconditioned MSCs change the composition of the secretome by modulating the microenvironment. These results suggested that injured brain extracts provide a specified microenvironment for MSCs, which enhanced the therapeutic effect of the secretome on TBI. Therefore, we conjectured that combining the injury-preconditioned secretome in a collagen–heparan sulfate scaffold may control the secretome release rate and reconstruct the neural network in the lesion area more effectively after TBI.

In order to mimic the complex microenvironment during TBI, brain tissue extracted from cerebral infarction was used to precondition HUCMSCs. We developed 3D-printed injury-preconditioned secretome/collagen/heparan sulfate scaffolds in an effort to determine the feasibility of scaffold implantation for repairing neural networks following TBI.

## Materials and methods

### Animals

Eight-week-old Sprague Dawley rats (male, 235 ± 15 g) were purchased from SPF Biotechnology Co. Ltd. (Beijing, China). All rats received humane care. The protocol was approved by the Research Animal Ethics Committee of People’s armed police (approval code 23,658/42). Throughout the trial, all rats were housed in a 12-h light/dark cycle with unrestricted access to food and water (24 ± 2 °C, 40% humidity). The present study was not preregistered. Study protocols were not preregistered. Randomization was performed with the online tool QuickCalcs from GraphPad. Rats were coded and assigned randomly to four groups (Sham, TBI, 3D-CH-ST, 3D-CH-IB-ST) using "Random numbers” in QuickCalcs. Rats were labeled by ear tagging method. This study was blinded. The specific blinding method was that the experimentalists were not aware of the group of animals during the experiment and statistical analysis. In addition, a different person performed the analysis or experimental group assignment. The analysis was performed using animal codes for blinded quantification. The experiments were then performed according to the order of the experimental groups. No sample calculation was performed. When the score was greater than 10, it indicated that the severe TBI model was successfully established. If the mNSS score was greater than 10 at 3 h after TBI, these rats were included. Rats were excluded if the mNSS score was less than 10. If the obtained values were above or below the mean ± 3σ, these values were excluded from the data. In the injury group, 4 rats died during TBI modeling (TBI group: 1, 3D-CH-ST group: 2, 3D-CH-IB-ST group: 1). Based on the exclusion criteria, 2 rats were excluded (TBI group: 1, 3D-CH-IB-ST group: 1). Within 3 days after TBI, 6 rats died in the injury group (rats in the TBI group: 2, 3D-CH-ST group: 3, 3D-CH-IB-ST group: 1). Throughout the experiment, the experimentalists took measures to reduce the number of animals and reduce their suffering. Wound hemostasis during surgery was achieved by local instillation of thrombin (50 ~ 1000 units/mL) (Solarbio, Beijing, China, Cat# T8021).

### Isolation, culture, and analysis of HUCMSCs

According to published accounts, HUCMSCs were isolated from umbilical cords [40]. Informed consent from umbilical cord donors could not be made publicly available due to hospital regulations. In brief, neonatal cords were washed with sterile saline and cut into 1 mm^3^ pieces. The patches were then digested in a mixture containing 0.2% hyaluronidase and 0.2% type II collagenase. After 3 h, the mixture was filtered through a 100-µm filter to obtain a cell suspension. Cells were cultured with culture medium, with a half-volume of medium change every 2 days.

### TBI model

The previously established method of lateral fluid percussion was utilized to create the rat model of TBI [39, 41]. In brief, rats were anesthetized with isoflurane inhalation (3% for induction, 1.5% for maintenance (v/v)) to ensure sufficient anesthesia during the whole procedure. In the right parietal area of the rat skull, a 5-mm bone window was produced using the stereotaxic device (2.1 mm posterior to the coronal suture and 3.8 mm lateral to the sagittal suture). The rats were then exposed to an impact injury using a controlled cortical impact device (parameters: depth 2 mm, impact velocity 5 m s^−1^, residence time 12 ms). The pain was relieved intramuscularly with tramadol (1 mg/kg) (Solarbio, Beijing, China) after surgery. After surgery, the rats were placed on warm pads until they awoke.

### Isolation and collection of the HUCMSCs secretome (ST) and injury-preconditioned secretome (IB-ST)

The secretome was obtained from HUCMSCs according to the existing methods [39]. 72 h after TBI induction, rats with significant hemiplegia symptoms and neurological deficits were screened out (modified Neurological Severity Score ≥ 10). Rats were anesthetized with an intraperitoneal dose of 1.3 percent pentobarbital sodium (40 mg/kg) and decapitated. The right cortex in the ipsilateral region of TBI rats was dissected on ice (area of injury plus a 2-mm penumbra around the impacted tissue or corresponding tissue in the control group). Following the addition of Dulbecco's Modified Eagle Medium (DMEM) at 150 mg/mL, magnetic beads were used to crush the tissue pieces. Centrifugation was performed at 10,000×*g* for 20 min at 4 °C, and a 0.22-μm filter was used to obtain brain tissue extracts. The supernatant was reserved at − 80 °C for the treatment of HUCMSCs.

For secretome production, HUCMSCs were incubated with DMEM containing 10% fetal bovine serum for 24 h. Then, the culture media was changed to DMEM containing injured brain extract. After incubating for 24 h, the culture medium was replaced with serum-free, low-glucose DMEM. After 24 h, the medium was collected and centrifuged at 500×*g* for 10 min, twice at 800×*g* for 15 min. The Minimate TFF capsule system (PALL Corporation, Ann Arbor, MI, USA) with a 100 kDa membrane was used to concentrate the supernatants by ultrafiltration. Subsequently, the secretome produced by 1 × 10^7^ cells was concentrated to 20 μL. The collected secretome consisted of the following two groups: ST (normal HUCMSCs secretome without treatment) and IB-ST (preconditioned HUCMSCs secretome with injured brain extract).

### Preparation of 3D-printed injury-preconditioned secretome/collagen/heparan sulfate scaffolds

Collagen/heparan sulfate (CH) composites were prepared as described previously [15, 23]. To create a collagen gel, the salt precipitate of collagen was dialyzed in deionized water at 4 °C for 5 days. In 50 mL of 0.05 M acetic acid solution, 3 g of collagen and 100 mg of heparan sulfate (Sigma, USA) were dissolved (Solarbio, Beijing). After complete mixing, the combined solution was exposed to UV radiation (365 nm, 18 W cm^−2^), which cross-linked the polymer, for 10 min. The solution containing 200 µg of ST or 200 µg of IB-ST was mixed with 0.1 g collagen-heparan sulfate mixed solution and incubated overnight after 12 h of stirring at 4 °C. The prepared mixed solution (ST/collagen/heparan sulfate compound material and IB-ST/collagen/heparan sulfate compound material) was placed in a printer cartridge. Preparation of 3D-printed ST/collagen/heparan sulfate scaffolds (3D-CH-ST) and 3D-printed IB-ST/collagen/heparan sulfate scaffolds (3D-CH-IB-ST) was performed at − 20 degrees using a 3D printer (Regenovo Biotechnology Co., Ltd., Hangzhou, China) according to a previously reported protocol [23]. The printing parameters were as follows: platform temperature = − 20 °C, printing speed = 100 mm min^−1^, layer thickness = 0.1 mm. After printing, 3D-CH-ST and 3D-CH-IB-ST were then freeze-dried for 48 h. The following four types of 3D-printed scaffolds were used in this study: 3D-printed ST/collagen scaffolds (3D-C-ST), 3D-printed IB-ST/collagen scaffolds (3D-C-IB-ST), 3D-CH-ST, and 3D-CH-IB-ST.

### Scanning electron microscopy (SEM)

In order to immobilize 3D-CH-ST scaffolds and 3D-CH-IB-ST scaffolds, they were first immersed in 2% glutaraldehyde (Aladdin Biotech, Shanghai) and 1% osmium tetroxide (Sigma, USA), followed by gradient acetone dehydration (Solarbio, Beijing). Using liquid nitrogen, the samples were quickly frozen to the required temperature before being freeze-dried for 12 h. The dried samples were then covered in gold and examined using a scanning electron microscope (Hitachi, Japan).

### The scaffold deterioration test conducted in vivo

In order to explore the suitable scaffold mass ratio, five 3D-CH-IB-ST scaffolds with different mass ratios were prepared (collagen/heparan sulfate = 10:1, 20:1, 30:1, 40:1, 50:1). In vivo degradation experiments of the scaffold were performed according to the defined method. Briefly, after 3% isoflurane anesthesia, the backs of the rats were incised to form an opening of approximately 1 cm. A scaffold of the same mass ratio was placed in the wound. One to eight weeks after surgery, scaffolds were removed from the backs of the rats to evaluate in vivo degradation.

### Features of the scaffolds' physical construction

The water absorption and porosity of the scaffolds were measured by the weight method and the volume method [23]. Three samples of each dried support were obtained and soaked in 0.01 mol L^−1^ PBS for 24 h at a pH of 7.4 to attain equilibrium in order to assess the water absorption. The weight of absorbed water on the surface was recorded as *m*0, and the mass after drying in the drying cabinet for 2 h was recorded as *m*1. The water absorption ratio was calculated by the following formula: water absorption (%) = (*m*0 − *m*1)/*m*1 × 100%. For porosity, anhydrous ethanol with a volume of *V*1 was removed with a measuring cylinder, and the dry scaffold sample was soaked for 5 min. When the ethanol was vacuolated until there were no bubbles, the volume of ethanol was recorded as *V*2. The scaffold was removed and the volume was recorded as *V*3. The porosity ratio was calculated as follows: the porosity ratio (%) = (*V*1 − *V*3)/(*V*2 − *V*3) × 100%. Instron 5865 material testing equipment (Instron, USA) was used to assess the mechanical qualities of the scaffolds. After reaching equilibrium, the scaffolds were submerged in 0.01 M PBS at 37 °C for 24 h. Three sets of samples were then taken, and the elastic modulus of compressive strain was determined. Three cycles of a 0.5 Hz sawtooth waveform with 0.1 N of preload applied in 50% increments and a speed of 100%/min were employed.

### Determination of the kinetics of secretome release from 3D-CH-ST

3D-CH-IB-ST and 3D-C-IB-ST were placed in PBS at 37 °C, and the supernatants were collected at 0, 5, 10, 15, 20, 25, and 30 days after incubation. The supernatant was then completely replaced with fresh PBS. The amount of secretome in the supernatant at each time point was analyzed by a BCA protein concentration assay kit.

### Cytocompatibility of scaffolds and cell staining

On 3D-CH-ST and 3D-CH-IB-ST, HUCMSCs were inoculated at a density of 1 × 10^6^/mL. Following a 7-day culture, inverted phase contrast microscopy, scanning electron microscopy (SEM), and HE staining were used to examine the development of the cells on the scaffolds. Each scaffold received 100 μL of HUCMSCs at a concentration of 1 × 10^6^/mL. Using the MTT technique (Solarbio, Beijing), the vitality of cells on 3D-CH-ST and 3D-CH-IB-ST at 1, 3, 5, and 7 days after culture was determined.

From the embryonic day 14 brain, neural stem cells (NSCs) were extracted and cultured in accordance with accepted practices [42]. Immunofluorescence staining for Nestin (1:200, Invitrogen, USA) was performed to identify NSCs. Similarly, NSCs were inoculated into the scaffold at a density of 1 × 10^6^/mL. Immunofluorescence staining of cell–scaffold complexes was performed after 7 days of culture of NSCs with scaffolds. The sections were permeabilized with 0.5% Triton X-100 and blocked with 10% normal goat serum (NGS). NSCs were injected onto the scaffold in a 20 μL single-cell solution at a concentration of 5 × 10^6^/mL. The cell adhesion rate of NSCs was determined at 1, 12, 24, 36, 48, 60, and 72 h after seeding using the formula cell adhesion rate = (number of adherent cells/number of seeded cells) × 100%. The MTT technique was used to assess the viability of NSCs on 3D-CH-ST and 3D-CH-IB-ST at 1, 3, 5, and 7 days after culture. After culture, immunofluorescence staining was performed to assess the effect of 3D-CH-ST and 3D-CH-IB-ST on NSCs differentiation [43, 44]. The primary antibodies were mouse-Nestin (1:200, Invitrogen, USA), rabbit-NF (1:300, Abcam, UK), rabbit-GAP43 (1:500, Invitrogen, USA), rabbit-Tuj-1 (1:500, Abcam, UK), and mouse-NeuN (1:1000, Abcam, UK).

### Scaffold implantation

Before scaffold implantation, cylindrical brain tissue with a diameter of 2 mm and a height of 2 mm was removed by using a mold. After adequate hemostasis, a cylindrical scaffold (2 mm in diameter and 2 mm in height) was implanted into the cavity. One hundred and twenty rats in the Sham group underwent a bone window craniotomy but were not affected in any way. Rats were randomly divided into the Sham group (*n* = 30), TBI group (TBI without any implantation, *n* = 30), 3D-CH-ST group (TBI with the implantation of 3D-CH-ST, *n* = 30), and 3D-CH-IB-ST group (TBI with the implantation of 3D-CH-IB-ST, *n* = 30). The wounds were tightly sutured, and the rats were then placed in warm cages to recover from anesthesia. After TBI surgery, tramadol (1 mg/kg) (Solarbio, YZ-171255) and sodium penicillin (15 mg/kg) (Solarbio, C8250) were administered to all rats to relieve pain and avoid infection. All experimental operations were performed from 8:00 am to 12:00 pm.

### Detection of neurological function

The modified neurological severity scores (mNSS) were performed at 1, 3, 7, 14, 21, and 28 days postinjury to evaluate the recovery of neurological function (*n* = 10 for each group). The mNSS was scored on an 18-point scale. Briefly, rats were trained and evaluated before surgery to confirm the normal score (0). The neurological function scores were also analyzed at different time points after injury.

At 2 months postoperatively, the amplitude and latency of motor evoked potentials (MEP) were recorded to assess the differences in locomotor function between the four groups due to damage to the motor cortex of the brain (*n* = 10 for each group). Two needle electrodes were fastened to the lateral side of the muscle, corresponding to the C1 and C2 areas of the human brain as stimulation electrodes. Needle recording electrodes were introduced into the muscular belly of the forelimb extensor muscle and the posterior tibial nerve of the hindlimb. To determine the MEP state of the extremities, continuous electrical stimulation was used. The following parameters were used for the electrophysiological analysis: stimulation frequency of 1 Hz, pulse width of 0.2 ms, and stimulation intensity of 46 V. An evoked potential device was used to capture the waveforms and data that were gathered.

The Morris water maze (MWM) test was performed from day 21 to day 26 after surgery to assess cognitive function (*n* = 10 for each group). Before training, the platform was placed in the northeast quadrant, and the rats were put into the pool facing the pool wall. The escape latency was defined as the time to enter the water to climb the platform. Successful training was considered when the rats located the platform, climbing upon it and staying for at least 2 s. Rats that failed to find the hidden platform within 60 s were guided to the location of the platform, and their escape latency was recorded as 60 s. Each rat was trained 4 times in total. The platform was removed 27 days after TBI, and the rats were subjected to cognitive testing in the form of a space searching experiment. The escape latency, number of platform crossings, and time spent in the target quadrant were recorded and analyzed.

### Histological observations

As previously mentioned, HE staining, Bielschowsky’s silver staining, Nissl staining, and immunofluorescence staining were carried out at 8 months after TBI (*n* = 5 for each group) [39, 45]. Pentobarbital sodium solution at a concentration of 1.3% (40 mg/kg) was injected intraperitoneally to anesthetize the rats. Cardiac perfusion was conducted by using normal saline and 4% paraformaldehyde successively. On the following day, dehydration was performed with 30% sucrose solution for another 5 days. Sections of brain tissues (20 μm thickness) were prepared in the coronal plane. The sections were permeabilized with 0.5% Triton X-100 and blocked with 10% normal goat serum (NGS). The primary antibodies were mouse-Nestin (1:200, Invitrogen, USA), rabbit-NF (1:300, Abcam, UK), rabbit-MBP (1:300, Abcam, UK), mouse-NeuN (1:1000, Abcam, UK), mouse-MAP-2 (1:250, Abcam, UK), rabbit-SYP (1:250, Invitrogen, USA), mouse-CD68 (1:100, Invitrogen, USA), rabbit-Iba1 (1:500, Invitrogen, USA), and rabbit-vWF (1:300, Invitrogen, USA). Sections were incubated with the primary antibody at 4 °C overnight before being incubated in the dark for 1 h at room temperature with the matching secondary antibody, either Alexa Fluor 488, Alexa Fluor 594, or Alexa Fluor 647. Sections were counterstained with DAPI. Two months after TBI, TUNEL staining was performed to detect apoptotic cells in the damaged area of TBI according to the established method [46]. The stained images with 400× magnification were selected for quantitative statistics. All quantitative statistical analyses of staining were carried out by ImageJ software (NIH, USA).

### Transmission electron microscopy (TEM)

Both 4% paraformaldehyde and 2% glutaraldehyde were injected into the aorta of each group of rats after they had been allowed to sleep with 1.2% sodium pentobarbital (40 mg/kg) anesthesia (*n* = 5 for each group). The injured brain tissue was separated and cut into cubes of 1 mm^3^ in size. Two percent glutaraldehyde and one percent osmium acid were used to fix the samples. The chunks of brain tissue were then desiccated and embedded in Epon 812 embedding medium (Solarbio, Beijing, China). A series of sections between 70 and 90 nm thickness were cut with an ultramicrotome, double-stained with lead and uranyl acetate, and then studied with a JEM 1210 TEM (JEM 1210; JEOL, Tokyo, Japan). Ten random field images of each sample at a magnification of 5000× magnification were used to measure the number of myelinated axons per 1000 m^2^, the diameter of myelinated axons, and the thickness of myelinated tissue [47]. Image-Pro Plus software (Media Cybernetics, USA) was implemented to perform quantitative analysis of TEM.

### Statistical analysis

GraphPad Prism 7.0 software (RRID: SCR_002798) was used to perform data processing and analysis. Data acquisition and analyses were blinded to the experimenter. One sample Kolmogorov‒Smirnov test was performed to determine the normal distribution for all data in this study. Measurement data are reported as the mean ± standard deviation. Statistical significance was determined by one-way analysis of variance (ANOVA) followed by Bonferroni analysis in multiple groups. Two-tailed Student’s *t* test was performed for pairwise comparisons. *P* < 0.05 was considered statistically significant.

## Results

### 3D-CH-IB-ST exhibited favorable physical properties and suitable biodegradability

**Fig. 1:**
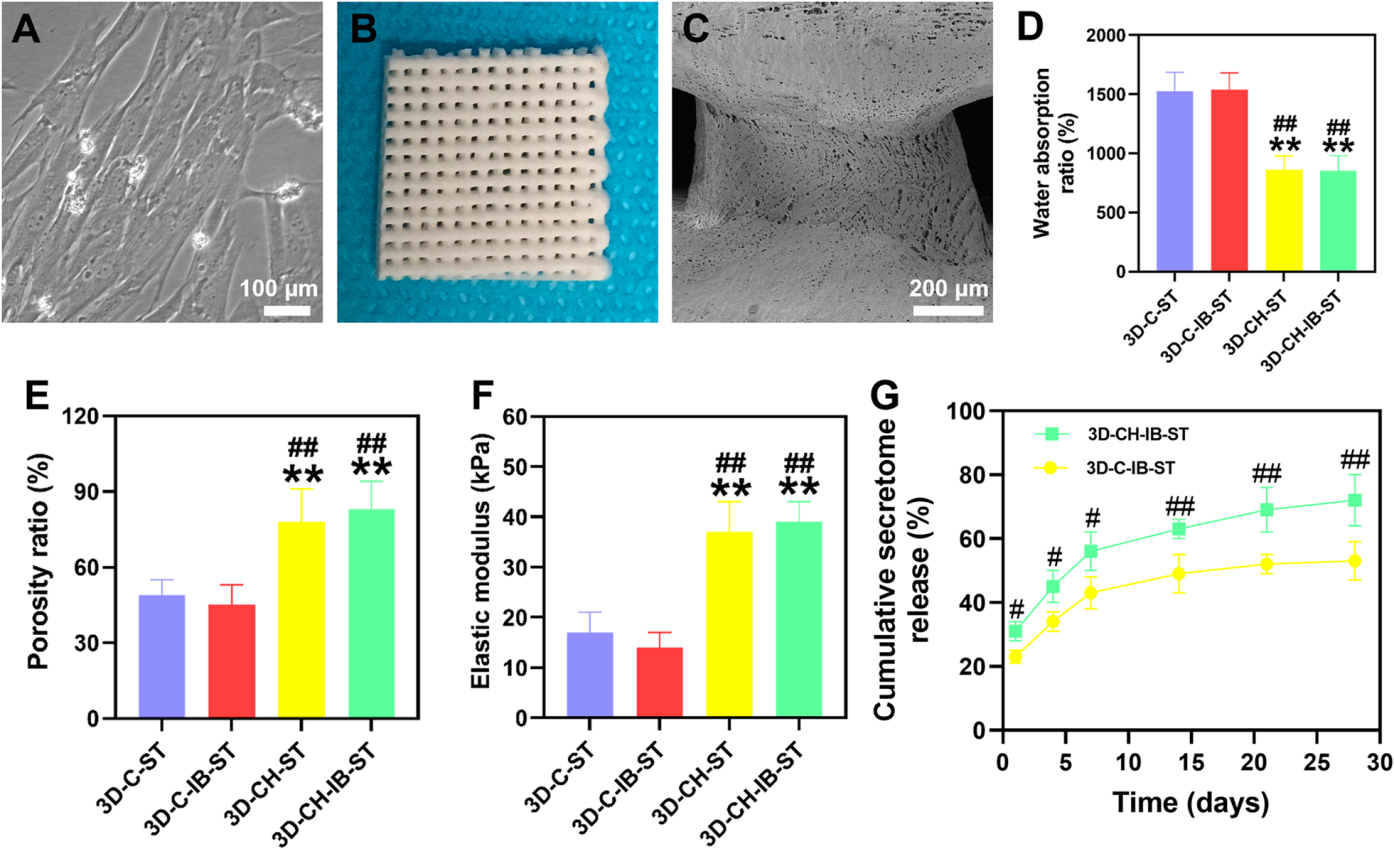
**A** Morphology of HUCMSCs under phase contrast microscopy. **B** General view of 3D-CH-IB-ST. **C** Typical representative images of TEM (**C**) of 3D-CH-IB-ST. **D**–**F** The water absorption (**D**), porosity ratio (**E**), and elastic modulus (**F**) of the 3D-C-ST and 3D-CH-IB-ST scaffolds. **G** Release profile of the secretome in 3D-C-IB-ST and 3D-CH-IB-ST for 30 days. ^**^P < 0.01 versus the 3D-C-ST group, ^#^*P* < 0.05, ^##^*P* < 0.01 versus the 3D-C-IB-ST group

HUCMSCs with fusiform morphology were examined using a phase-contrast microscope (Fig. 1A). 3D-CH-IB-ST was prepared by a 3D printer (Fig. 1B). The porous structure of 3D-CH-IB-ST was shown by electron microscopy and HE images, which offered a favorable environment for cell attachment and proliferation (Fig. 1C and Additional file 1: Fig. S1A–F, ESI). Different degradations were observed for 5 mass ratios of scaffolds. 8 weeks after implantation, 3D-CH-IB-ST scaffolds with 2 of 5 mass ratios (collagen/heparan sulfate = 20:1, 10:1) had not been completely degraded. Two of the 5 mass ratios (collagen/heparan sulfate = 50:1, 40:1) of the 3D-CH-IB-ST scaffold had been completely degraded within 4 weeks (Additional file 1: Fig. S1G, ESI). These findings showed that the scaffolds' degradation rate increased as a mass ratio of collagen to heparan sulfate increased. The degradation of the scaffold with the mass ratio of collagen/heparan sulfate = 30:1 was applicable to TBI repair process. Therefore, we chose the 3D-CH-IB-ST scaffold with a mass ratio (collagen/heparan sulfate = 30:1) for subsequent experiments. The water absorption of 3D-CH-IB-ST (853.12 ± 128.317) was lower than that of 3D-C-IB-ST (1537.26 ± 143.116) and 3D-C-ST (1528.08 ± 158.238) (*P* < 0.01), which suggested that the addition of heparan sulfate facilitated the maintenance of the secretome (Fig. 1D). In comparison with 3D-C-IB-ST (45.58 ± 8.368) and 3D-C-ST (49.36 ± 6.103), 3D-CH-IB-ST (83.28 ± 11.178) had a larger porosity (*P* < 0.01), which aided in the diffusion of the nutritional solution (Fig. 1E). The elastic modulus of 3D-CH-ST (37.53 ± 6.373) and 3D-CH-IB-ST (39.17 ± 4.452) was significantly higher than that of 3D-C-ST (17.68 ± 4.252) (*P* < 0.01), which supported tissue regeneration (Fig. 1F). A higher and more stable release was observed from day 1 to day 30 in the 3D-CH-IB-ST group than in the 3D-C-IB-ST group, which ensured that the secretome played a beneficial role in vivo (Fig. 1G).

### 3D-CH-IB-ST exhibited good cytocompatibility in vitro

**Fig. 2:**
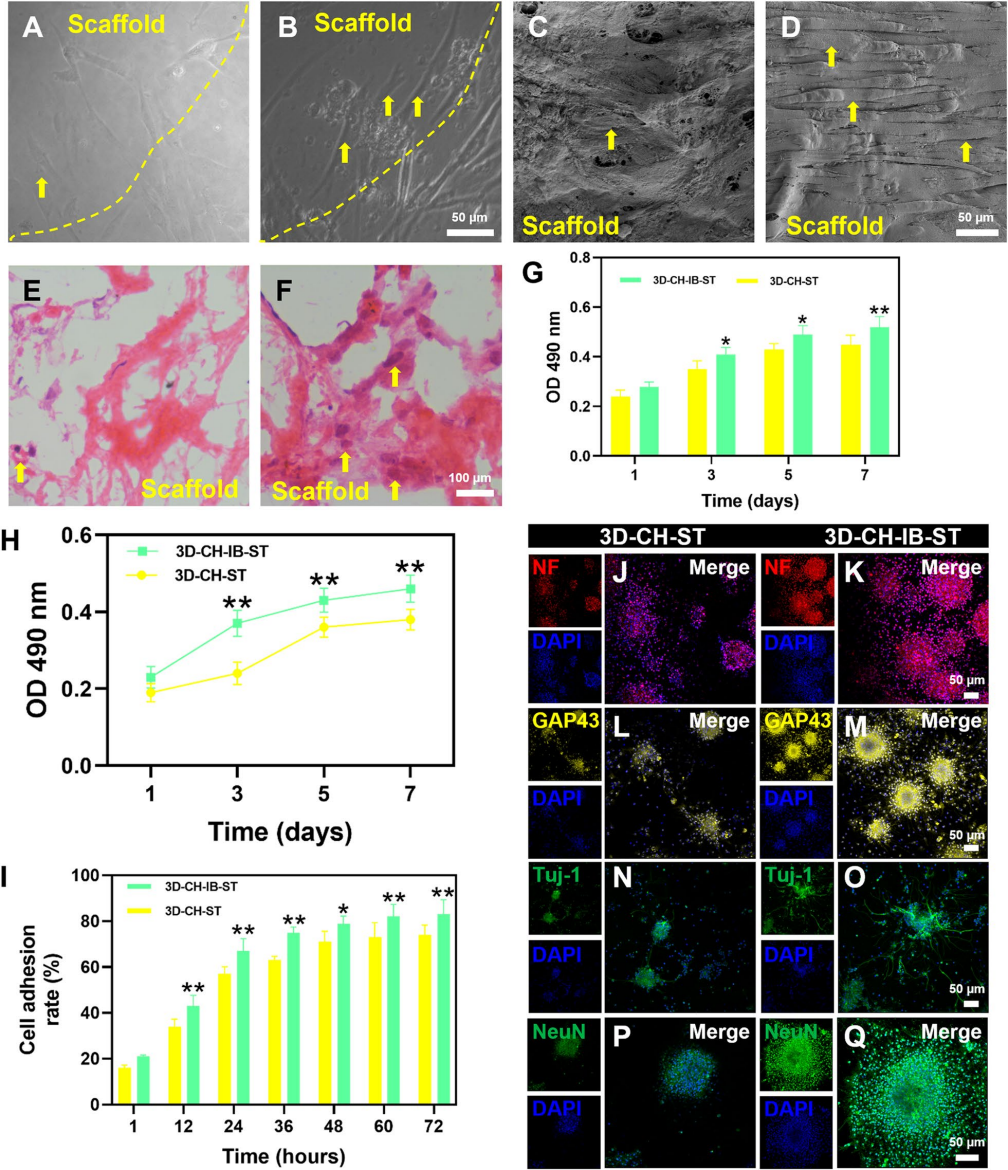
**A**–**F** Representative images of HUCMSCs cultured with 3D-CH-IB-ST scaffolds by phase contrast microscopy (**A**, **B**), SEM (**C**, **D**) and HE staining (**E**, **F**). **G** MTT analysis was performed after 1, 3, 5, and 7 days of HUCMSC cultured with 3D-CH-IB-ST. **H**, **I** MTT analysis **H** and cell adhesion rate **I** of NSCs cultured with 3D-CH-IB-ST. **J**–**Q** Immunofluorescence staining of NF, GAP43, Tuj-1, and NeuN in NSCs cultured with 3D-CH-IB-ST. ^*^P < 0.05, ^**^P < 0.01 versus 3D-CH-ST group

After 7 days of culture, phase contrast microscopy, SEM, and HE staining showed that HUCMSCs had excellent cell morphology and grew on the scaffolds' surface and within their pores (Fig. 2A–F). The light absorbance of HUCMSCs in the 3D-CH-IB-ST group was significantly increased at 3, 5, and 7 days compared to that in the 3D-CH-ST group (*P* < 0.05) (Fig. 2G). NSCs cultured in vitro were observed under a phase contrast microscope (Additional file 1: Fig. S2A, ESI). The immunofluorescence results showed Nestin expression in the neurospheres (Additional file 1: Fig. S2B–D, ESI). At 3, 5, and 7 days after culture, the higher light absorbance of NSCs in the 3D-CH-IB-ST group was observed (*P* < 0.01) (Fig. 2H). The cell adhesion rate of NSCs in the 3D-CH-IB-ST group was noticeably higher than that of the 3D-CH-ST group at the same time (*P* < 0.05) (Fig. 2I). The results of MTT showed that 3D-CH-IB-ST had better biocompatibility and that the 3D-CH-IB-ST group was more favorable for cell survival than the 3D-CH-ST group.

### 3D-CH-IB-ST promoted axonal growth and upregulated primary and mature neuronal markers in NSCs in vitro

To further investigate the potential mechanism by which 3D-CH-IB-ST promotes the growth of NSCs, we examined the expression of the axon-associated markers neurofilament (NF) and growth-associated protein-43 (GAP43) and the expression of neuron-associated markers (primary neuronal marker: Tuj-1; mature neuron marker: NeuN). Seven days after culture, the expression of NF, GAP43 and Tuj-1 was significantly higher in the 3D-CH-IB-ST group (Fig. 2J–O), suggesting that the inclusion of the injured brain-secretory contributes to axonal growth and the formation of primary neurons. Fourteen days after culture, the expression of NeuN in the 3D-CH-IB-ST group was also markedly increased compared with that in the 3D-CH-ST group (Fig. 2P–Q), which indicated that 3D-CH-IB-ST was more effective in promoting neuronal maturation.

### 3D-CH-IB-ST implantation ameliorated neurological function recovery after TBI

**Fig. 3:**
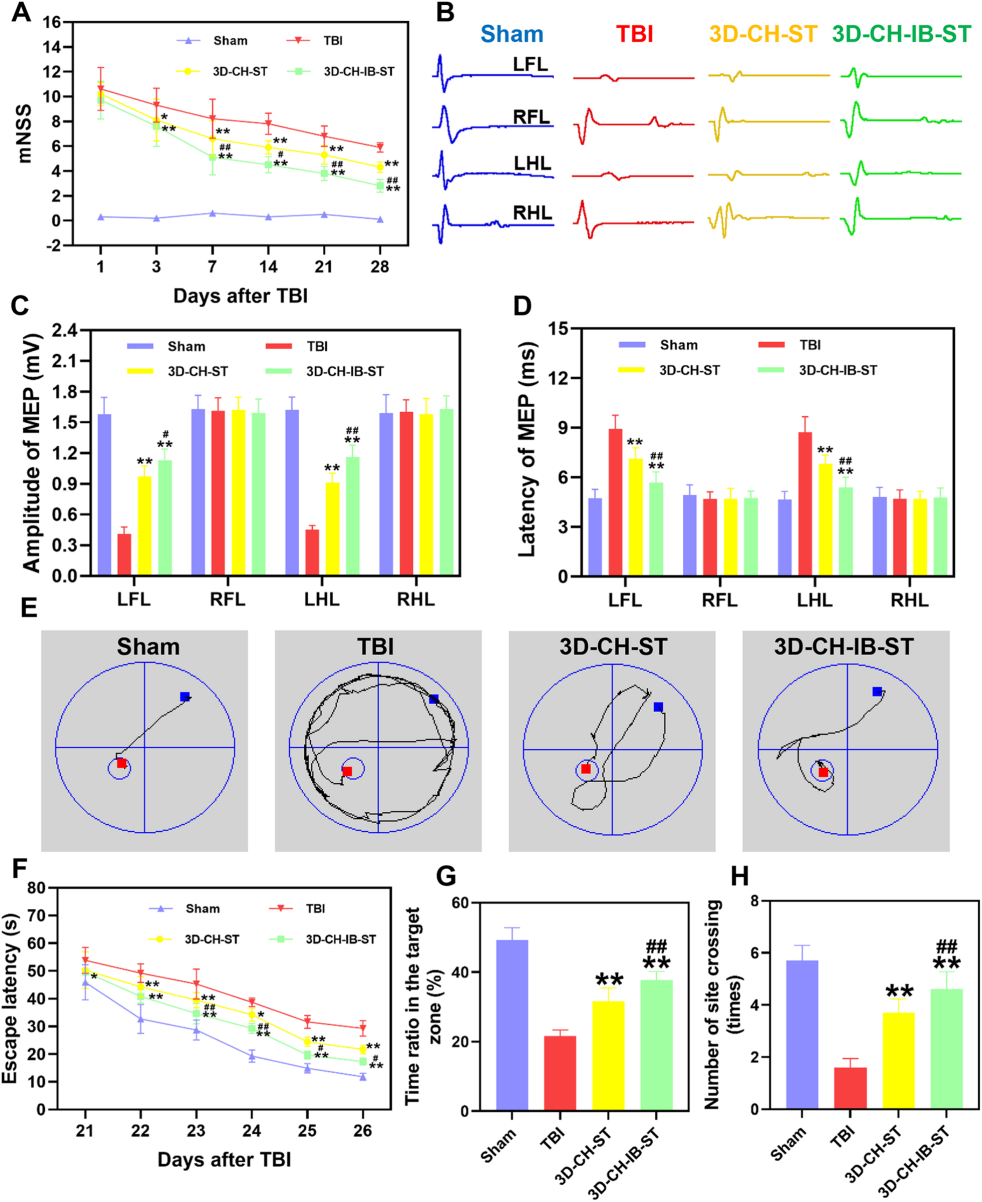
**A** Rat mNSS scores were recorded at 1, 3, 7, 14, 21, and 28 days after implantation of differentiated scaffolds after TBI. **B** Representative motor evoked potential waveforms at 2 months posttransplant in the four groups. **C**, **D** The amplitude (**C**) and latency (**D**) of MEP in four groups. **E** Representative swimming paths in the Morris water maze in the four groups. **F**–**H** Escape latency (**F**), time spent in the target quadrant (**G**), and number of site crossings (**H**) in the four groups. ^*^P < 0.05, ^**^P < 0.01 versus TBI group, ^#^*P* < 0.05, ^##^*P* < 0.01 versus 3D-CH-ST group

We then investigated whether 3D-CH-IB-ST implantation might enhance locomotor function recovery and repair brain injury. At 1 day after TBI, all injured rats exhibited increased mNSS scores (Fig. 3A). Decreased mNSS scores were found in the 3D-CH-ST and 3D-CH-IB-ST groups compared to the TBI group (Fig. 3A). Notably, 3D-CH-IB-ST treatment recovered neurological function to a higher level than 3D-CH-ST treatment (Fig. 3A). Electrophysiological experiments showed that the 3D-CH-IB-ST group exhibited better recovery of locomotor function than the 3D-CH-ST group (Fig. 2B–D). The number of stimulated axons and the rate of nerve conduction are often reflected in the amplitude and latency of the MEP. Compared with the TBI group, both 3D-CH-ST and 3D-CH-IB-ST partially restored the normal electrophysiological waveforms of the left hind limb and left forelimb at 2 months after TBI (Fig. 3B). The 3D-CH-IB-ST group (the left forelimb: amplitude 1.13 ± 0.108, latency 5.68 ± 0.653; the left hindlimb: amplitude1.16 ± 0.116, latency 5.39 ± 0.612) exhibited significantly improved amplitude and latency of the left hindlimb and left forelimb, and the effect was superior to that of the TBI group (the left forelimb: amplitude 0.41 ± 0.068, latency 8.93 ± 0.826; the left hindlimb: amplitude 0.45 ± 0.0426, latency 8.73 ± 0.937) and the 3D-CH-ST group (the left forelimb: amplitude 0.97 ± 0.103, latency 7.13 ± 0.658; the left hindlimb: amplitude 0.91 ± 0.0937, latency 6.82 ± 0.529) at 2 months after TBI (Fig. 3C, D). The Morris water maze test was used to assess whether 3D-CH-IB-ST could impart any cognitive benefits. There is an overall improvement in spatial memory ability in TBI rats after 3D-CH-IB-ST treatment with shortening of routes (Fig. 3E). The 3D-CH-IB-ST group promoted cognitive function by decreasing latency and increasing the time spent in the target quadrant and the number of platform crossings compared with the TBI group. Notably, the 3D-CH-IB-ST group showed shorter latency, more time spent in the target quadrant and more platform crossings than the 3D-CH-ST group (Fig. 3F–H).

### 3D-CH-IB-ST implantation significantly improved nerve regeneration

**Fig. 4:**
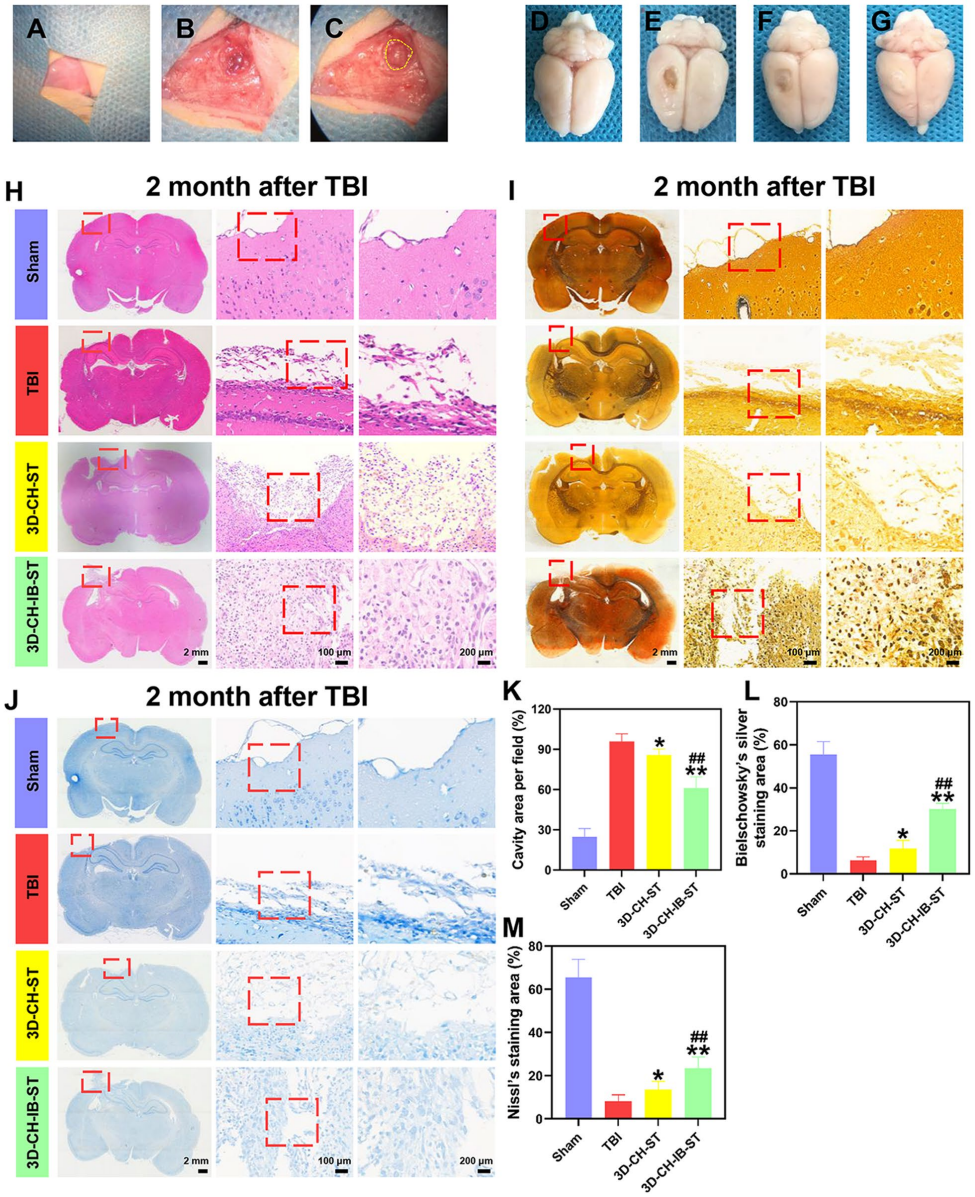
**A**–**C** Flowchart of 3D-CH-IB-ST implantation. **D**–**G** Representative general views at 2 months after implantation in the four groups. **H**–**J** Representative images of HE staining (**H**), Bielschowsky’s silver staining (**I**), and Nissl staining (**J**) at 2 months after implantation in the four groups. **K**–**M** Statistical calculation of the cavity area (**K**), Bielschowsky’s silver staining (**L**), and Nissl staining area (**M**) in the four groups. ^*^P < 0.05, ^**^P < 0.01 versus TBI group, ^##^*P* < 0.01 *versus* 3D-CH-ST group

To further examine the effect of 3D-CH-IB-ST on TBI treatment, histological staining was performed on brain tissue sections at 2 months after TBI. The procedure of damaged brain tissue removal and scaffold implantation is briefly shown (Fig. 4A–C). A general view of brain tissue showed that the loss volume was significantly decreased in the 3D-CH-IB-ST group compared to the 3D-CH-ST and TBI groups (Fig. 4D–G). HE staining showed that the TBI group had fewer new cells at the injury site, the nuclei of the nerves around the lesion crinkled, and the cells were deformed (Fig. 4H). In the 3D-CH-ST and 3D-CH-IB-ST groups, newborn cells were found at the site of injury (Fig. 4H). Compared to the TBI group (95.84 ± 5.663) and 3D-CH-ST group (85.73 ± 4.536), 3D-CH-IB-ST (61.13 ± 8.326) implantation could significantly decrease cavity area (*P* < 0.01) (Fig. 4H, K). The above results suggested that 3D-CH-IB-ST implantation had a facilitative effect on the regeneration of the lesion area after TBI.

In comparison with the TBI (6.32 ± 1.626) and 3D-CH-ST (11.81 ± 3.863) groups, Bielschowsky’s silver staining in the 3D-CH-IB-ST group (30.31 ± 2.538) showed a considerable increase in nerve fibers and new neurons in the region of injury (Fig. 4I, L). Nissl staining also underwent similar modifications, with 3D-CH-IB-ST (23.43 ± 5.243) showing a larger density and broader dispersion of Nissl bodies than the TBI (8.32 ± 2.831) and 3D-CH-ST (13.61 ± 3.758) groups (Fig. 4J, M). These results suggest that implantation of 3D-CH-IB-ST after TBI was helpful in restoring nerve fibers and neurons.

**Fig. 5:**
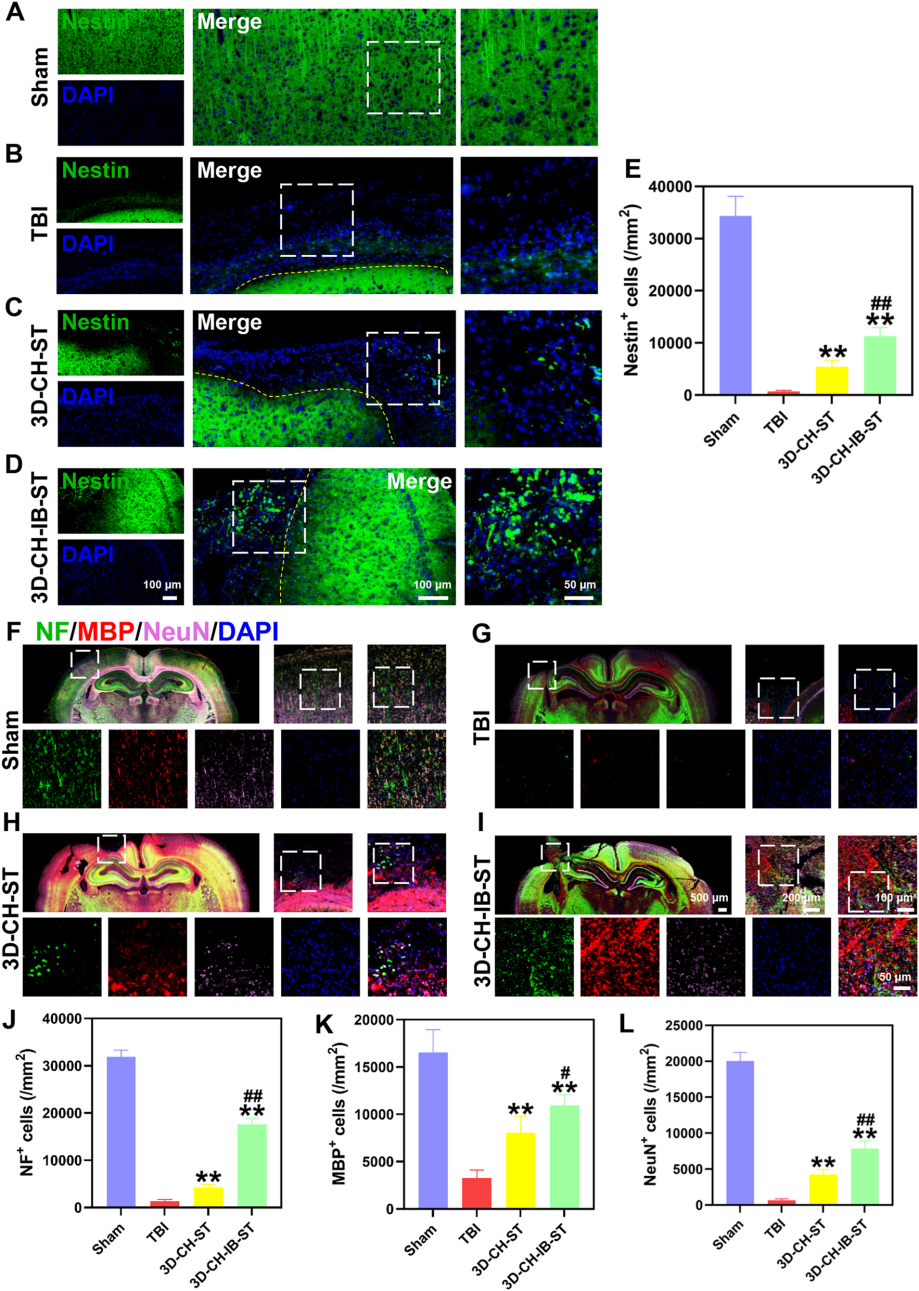
**A**–**D** The expression of Nestin around the injury site after TBI in the four groups. **E** Statistical analysis of Nestin^+^ cell numbers. **F**–**I** The expression of NF, MBP, and NeuN around the injury site after TBI in the four groups. **J**–**L** Statistical analysis of NF^+^ (**J**), MBP^+^ (**K**), and NeuN^+^ (**L**) cell numbers. ^**^P < 0.01 versus TBI group, ^#^*P* < 0.05, ^##^*P* < 0.01 versus 3D-CH-ST group

**Fig. 6:**
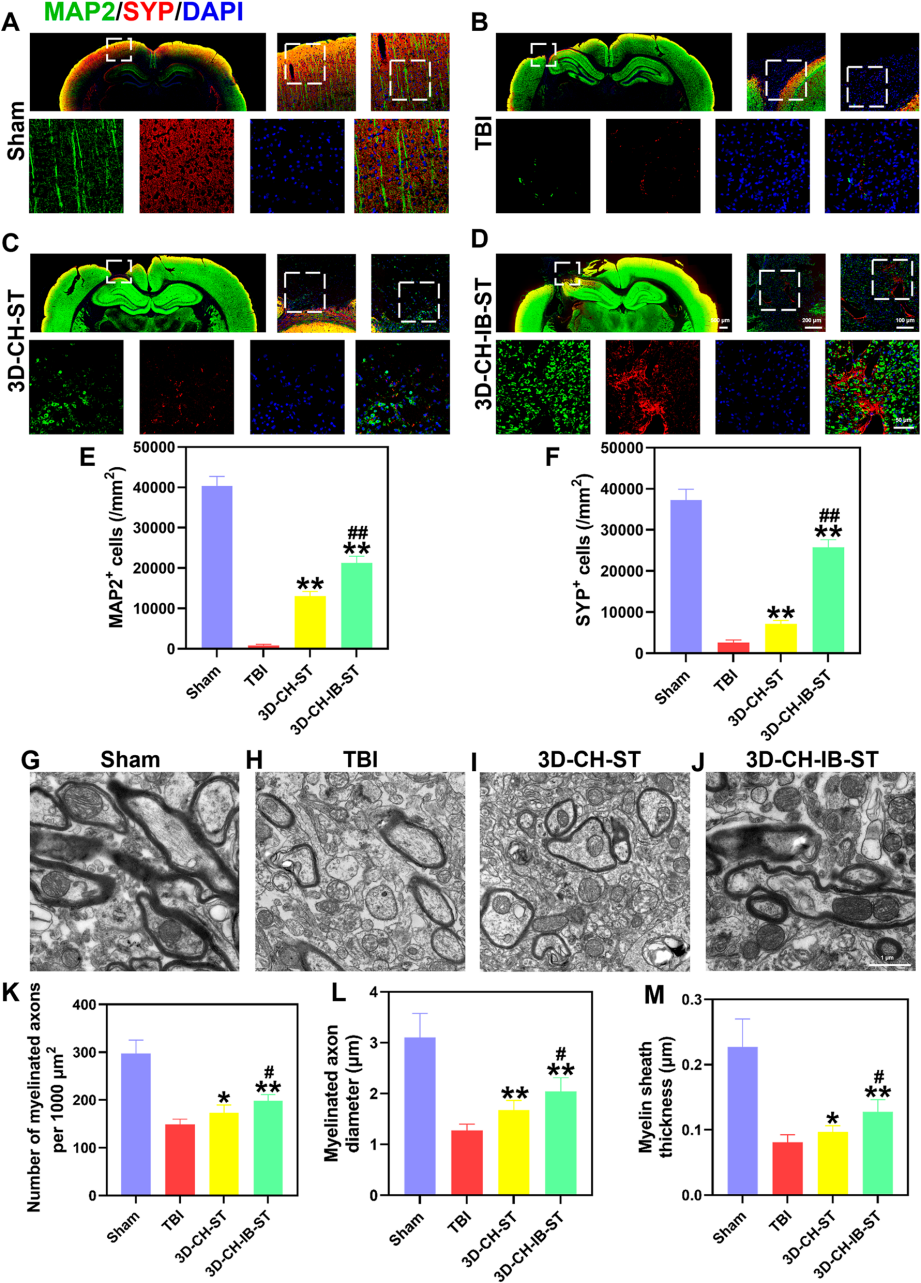
**A**–**D** The expression of MAP2 and SYP around the injury site after TBI in the four groups. **E**, **F** Statistical analysis of MAP2^+^ (**E**) and SYP^+^ (**F**) cell numbers. **G**–**J** Representative TEM images of the four groups. **K**–**M** Statistical analysis of the number of myelinated axons per 1000 μm^2^ (**K**), myelinated axon diameter (**L**), and myelin sheath thickness (**M**) in the four groups. ^*^P < 0.05, ^**^P < 0.01 versus TBI group, ^#^*P* < 0.05, ^##^*P* < 0.01 versus 3D-CH-ST group

The immunofluorescence results showed that the 3D-CH-IB-ST group (11,324.31 ± 1618.562) expressed more Nestin than the TBI (711.76 ± 208.357) and 3D-CH-ST (5406.87 ± 1266.283) groups, which demonstrated that 3D-CD-IB-ST treatment recruited endogenous NSCs (Fig. 5A–E). Triple immunofluorescence labeling with NF, MBP, and NeuN was performed to examine the nerve fibers, myelin sheath, and mature neurons in the injury site after TBI. More NF^+^, MBP^+^, and NeuN^+^ cells were found in the 3D-CH-IB-ST group (NF^+^ 17,593.48 ± 1278.613; MBP^+^ 10,938.95 ± 1139.537; NeuN^+^ 7874.44 ± 1058.616) than in the TBI (NF^+^ 1361.85 ± 368.119;MBP^+^ 3276.79 ± 853.261; NeuN^+^ 684.70 ± 178.232) and 3D-CH-ST (NF^+^ 4250.75 ± 623.317; MBP^+^ 8069.72 ± 1762.134; NeuN^+^ 4254.94 ± 683.452) groups (Fig. 5F–L). Similar changes were noted in MAP2/SYP double immunofluorescence staining (Fig. 6A–F). These results suggested that 3D-CH-IB-ST implantation significantly increased nerve fibers, myelin sheaths, mature neurons, and synapses in the injured area of TBI (Fig. 5F–L and Fig. 6A–F).

For TEM analysis, representative ultrastructural micrographs of the injured region after TBI are shown in Fig. 6G–J. We examined the changes in myelin, and the number of myelinated axons per 1000 μm^2^, myelinated axon diameter, and myelin sheath thickness in the 3D-CH-IB-ST group (the number of myelinated axons per 1000 μm^2^ 198.41 ± 13.513; myelinated axon diameter 2.04 ± 0.27; myelin sheath thickness 0.13 ± 0.018) were significantly increased compared to those in the TBI (the number of myelinated axons per 1000 μm^2^ 148.81 ± 11.718; myelinated axon diameter 1.28 ± 0.124; myelin sheath thickness 0.08 ± 0.012) and 3D-CH-ST (the number of myelinated axons per 1000 μm^2^ 173.61 ± 16.826; myelinated axon diameter 1.67 ± 0.195; myelin sheath thickness 0.10 ± 0.009) groups (Fig. 6K–M). The better recovery of ultrastructure after TBI further indicated that implantation of 3D-CH-IB-ST could improve the remodeling of axons and myelin sheaths.

### 3D-CH-IB-ST implantation promoted vascular reconstruction in the lesion area after TBI

**Fig. 7:**
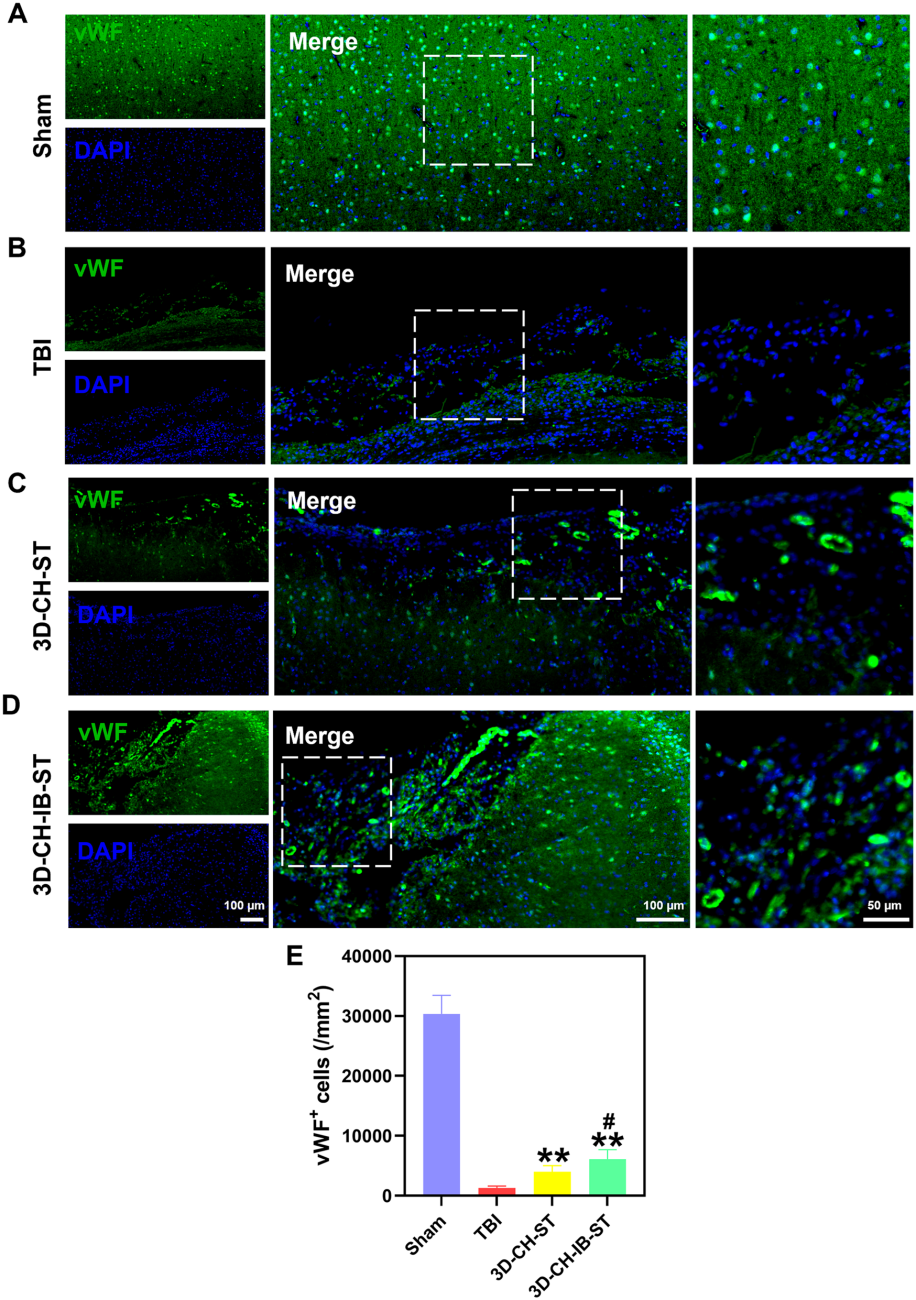
**A**–**D** vWF expression around the injury site after TBI in the four groups. **E** Statistical analysis of vWF^+^ cell numbers. ^**^P < 0.01 versus TBI group, ^#^*P* < 0.05 versus 3D-CH-ST group

vWF immunofluorescence staining was performed to detect vascular reconstruction in the lesion area after TBI. Few visible vWF-positive vessels were observed in the TBI group. Compared with the TBI group (1268.90 ± 356.218), more cells positive for vWF were observed in the 3D-CH-ST (3993.76 ± 1034.637) and 3D-CH-IB-ST (6069.02 ± 1628.827) groups (Fig. 7A–E). Additionally, the degree of vascular reconstruction in the 3D-CH-IB-ST group was relatively higher than that in the 3D-CH-ST group (Fig. 7A–E).

### 3D-CH-IB-ST implantation lessens inflammatory reactions and apoptosis after TBI

**Fig. 8:**
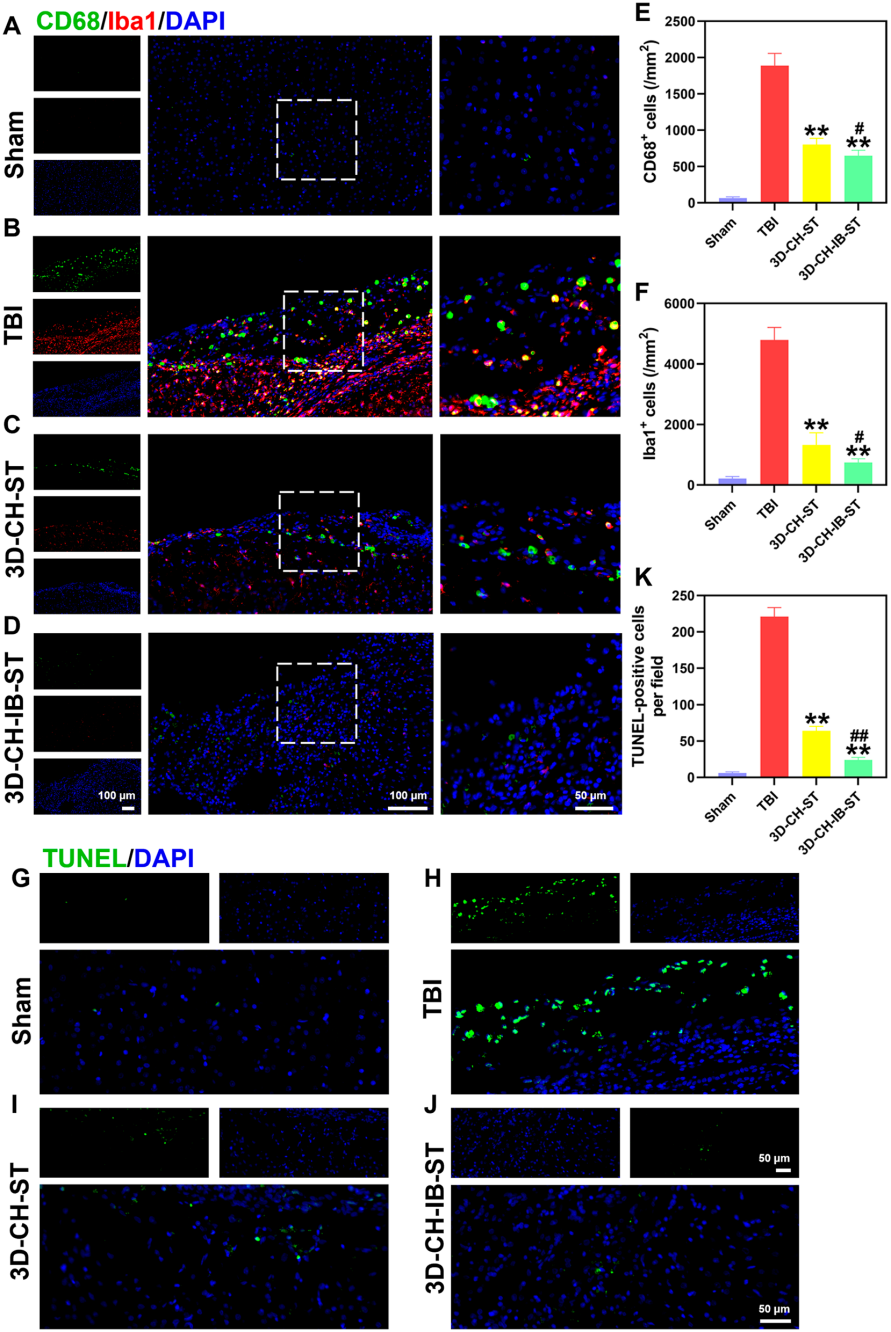
**A**–**D** CD68/Iba1 expression around the injury site after TBI in the four groups. **E**, **F** Quantitative analysis of CD68^+^ (**E**) and Iba1^+^ (**F**) cell numbers. **G**–**J** Representative TUNEL staining in the four groups. Apoptotic cells labeled with TUNEL emitted green fluorescence. **K** Quantitative counting of TUNEL-positive cells. ^**^P < 0.01 versus TBI group, ^#^*P* < 0.05, ^##^*P* < 0.01 versus 3D-CH-ST group

In order to detect inflammation levels and apoptosis in the lesion area after 3D-CH-IB-ST implantation, CD68/Iba1 double immunofluorescence staining and TUNEL staining were applied. CD68^+^/Iba1^+^ cells showed significantly higher expression after TBI, while 3D-CH-IB-ST (CD68^+^ 649.91 ± 73.283; Iba1 749.91 ± 116.346) treatment significantly decreased the inflammatory response in the lesion area compared to that in the TBI (CD68^+^ 1891.09 ± 167.13; Iba1 4792.92 ± 413.253) and 3D-CH-ST (CD68^+^ 803.19 ± 86.113; Iba1 1320.50 ± 406.118) groups (Fig. 8A–F). Compared with the Sham group, TBI induction led to brain injury with a substantial increase in TUNEL-positive cells. The number of apoptotic cells was decreased when 3D-CH-IB-ST (24 ± 3.587) was applied compared to the TBI (221.28 ± 12.538) and 3D-CH-ST (64.13 ± 6.341) groups (Fig. 8G–K).

### 3D-CH-IB-ST exhibited good biocompatibilityin vivo

**Fig. 9:**
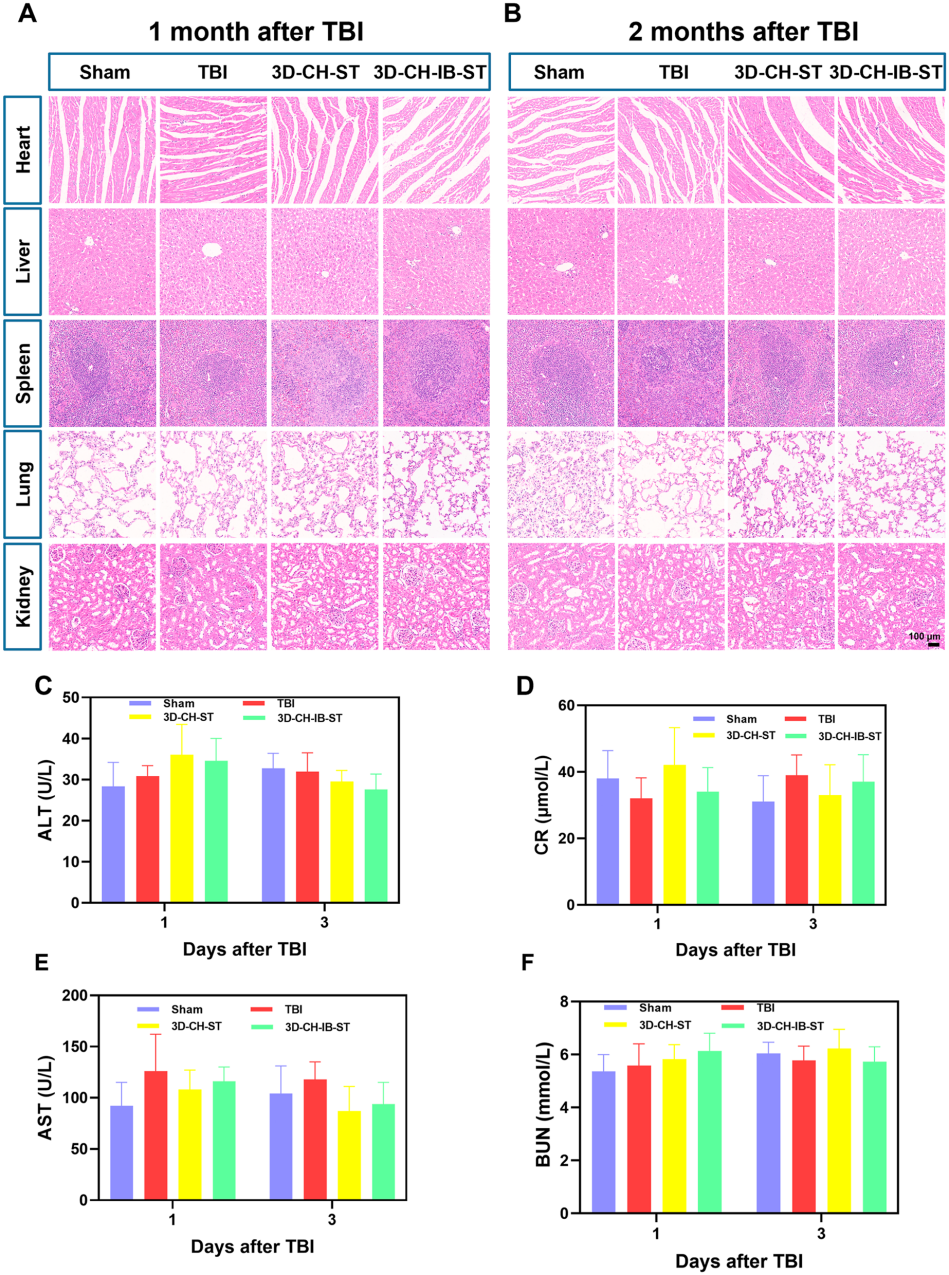
**A**, **B** Representative HE staining images of major organs at 1 and 2 months after TBI. **C**–**F** The levels of ALT, CR, AST, and BUN in plasma at 1 and 3 days after TBI

To confirm the feasibility of 3D-CH-IB-ST as a treatment for TBI, we further examined the tolerability of exposure to 3D-CH-IB-ST in vivo. Compared with the Sham group, no significant physiological abnormalities were seen in the major organs, such as the heart, liver, spleen, lung, and kidney by HE staining at 1 and 2 months after TBI (Fig. 9A, B). Further liver and kidney function indicators, such as alanine transaminase (ALT), creatinine (CR), aspartate aminotransferase (AST), and blood urea nitrogen (BUN), did not deviate from normal levels at 1 and 3 days after TBI (Fig. 9C, F). These results indicated that 3D-CH-IB-ST is biocompatible and has no toxic effects on the body, making it an ideal choice for TBI treatment.

## Discussion

High death and disability rates of TBI seriously endanger both human health and society [48, 49]. TBI often leads to neuronal and glial cell death, microvascular rupture, and axonal disruption, resulting in loss of sensorimotor and cognitive function [50]. Due to the limited ability of the CNS to repair itself, clinical treatments for TBI have difficulty achieving the goal of reconstructing neural networks. Over time, various biomaterials have been shown to have a significant ability to promote neural repair in different animal models of brain injury [51, 52]. Although the research of biomaterials has advanced significantly to this point, there are still no uniform selection criteria for materials. In this study, we chose collagen and heparan sulfate as scaffold bodies because they are both natural and have low immunogenicity. In addition, the addition of heparan sulfate significantly improved the physical properties of the scaffold and made it easier to load biologic factors.

In a previous study, we found that scaffolds prepared by cross-linking collagen and heparan sulfate had good physical properties and promoted recovery of locomotor function after TBI in canines [23]. Similarly, in this study, we demonstrated that collagen/heparan sulfate had good mechanical properties as a major part of the scaffold and could effectively load the injury-preconditioned secretome. We found that the mass ratio of 30:1 (collagen/heparan sulfate) degraded in vitro after 6 weeks. In addition, the secretome release assay demonstrated that heparan sulfate cross-linking significantly increased the release of the secretome in the scaffold, which made it suitable for treating TBI.

The ideal bioscaffold should have good biocompatibility, appropriate porosity, and pore connectivity [53, 54]. The scaffold had many uniform microporous structures, as shown by HE staining and TEM. Good connectivity between the microporous holes facilitated the migration of neural cells. The scaffolds loaded with injury-preconditioned secretome (3D-CH-IB-ST) significantly promoted the viability and adhesion of HUCMSCs and NSCs. We also demonstrated with in vivo experiments that 3D-CH-IB-ST had good biocompatibility and nontoxic effects on the body, making it ideal for the treatment of TBI.

The findings clearly showed that the secretome released from the scaffolds plays a crucial role at the location of the lesion after TBI. First, 3D-CH-IB-ST significantly promoted the differentiation and maturation of NSCs cultured in vitro. Then, we implanted 3D-CH-IB-ST into the cortical lesion area of rats after TBI, and mNSS scores were significantly improved. The MEP assay provided a more refined assessment of limb locomotor function and to some extent reflected the prognosis of TBI [55]. Significant improvements in the amplitude and latency of MEP in the left forelimb and left hindlimb suggested recovery of locomotor function in rats after implantation of 3D-CH-IB-ST. To further investigate the effect of 3D-CH-IB-ST on cognitive function, Morris water maze experiments were applied to rats. As we expected, the recovery of each indicator in the 3D-CH-IB-ST group was better than that in the 3D-CH-ST group, indicating that the injury-preconditioned secretome promoted the recovery of cognitive function after TBI. In this study, the injury-preconditioned secretome was involved in the pathophysiological process after brain injury and provided a specific microenvironment that accelerated the neurological repair effect of 3D-CH-IB-ST.

Therefore, it is necessary to investigate the potential contribution of 3D-CH-IB-ST to the recovery of neurological function after TBI. The results of several histological staining (HE staining, Bielschowsky’s silver staining, and Nissl staining) suggested that the 3D-CH-IB-ST group exhibited significantly enhanced recovery of brain tissue. By further immunofluorescence staining, we inferred a possible reason for 3D-CH-IB-ST to promote functional recovery. Differential expression of Nestin suggested that the 3D-CH-IB-ST group adequately recruited endogenous NSCs. The overall elevation of NF/MBP/NeuN in the 3D-CH-IB-ST group suggested that axonal and myelin structures were repaired and that endogenous neural stem cells matured at the site of lesion. The apparent increase in the number of MAP2^+^/SYP^+^ cells in the 3D-CH-IB-ST group suggested that these mature neurons already exhibited functional synapses. The cell number of the above indexes in the 3D-CH-IB-ST group was significantly increased compared to that in the other groups, indicating that the structural reconstruction of brain tissue was consistent with functional recovery. Neuronal myelin repair is essential for neural network reconstruction [56–58]. Improvement in myelin ultrastructure in the 3D-CH-IB-ST group was observed by TEM. By summarizing the above results, we inferred that 3D-CH-IB-ST promoted the recovery of neural function by inducing endogenous NSCs recruitment, differentiation, maturation, and axon and myelin sheath reconstruction.

3D-CH-IB-ST significantly promoted neural network reconstruction and neural function recovery benefiting from the richness of functional proteins and growth factors in the preconditioned secretome. Several studies have demonstrated that growth factors in the secretome have positive effects on neuronal survival, differentiation, and axonal growth [35, 59, 60]. Our previous study performed a high-density protein array of the injury-preconditioned secretome. The results showed that IB-ST contained 96 of these out of 174 key signaling proteins and 11 neurogene-related proteins (CNTF, NGF, EGF, BDNF, GDNF, IGF-1, NT-3, HGF, VEGF, bFGF, PDGF-AA) were significantly different compared to the untreated secretome [39]. Neural-specific factors in the injury-preconditioned secretome collaboratively participate in the neural reconstruction process. As mentioned earlier, the therapeutic effect of the secretome on TBI was attributed to multiple factors rather than a single factor. In addition, apoptosis suppressors, immunomodulatory factors, and angiogenic factors produced by the injury-preconditioned secretome also amplified the therapeutic effect of 3D-CH-IB-ST in TBI. Similarly, our findings demonstrated that 3D-CH-IB-ST significantly promoted revascularization and downregulated neuroinflammation and apoptotic responses in the lesion region.

In our research, there are still certain areas that want improvement. Because the body structure of non-human primates is closer to that of humans, 3D-CH-IB-ST needs to be implanted in the TBI model of non-human primates to evaluate the effect of 3D-CH-IB-ST on TBI repair before clinical trials. It is worthwhile to perform further research on the modification of chemical cross-linking and the enhancement of physical qualities in the creation of scaffolds [61–66]. The molecular mechanism by which 3D-CH-IB-ST promotes neural reconstruction after TBI is still unclear and needs to be further explored. In addition, we should be cautious about the suitability of this scaffold for humans.

## Conclusion

In summary, our findings confirmed that 3D-printed injury-preconditioned secretome/collagen/heparan sulfate scaffolds had good mechanical properties and biocompatibility. 3D-CH-IB-ST implantation significantly promoted neural network reconstruction and neurological recovery after TBI in rats.

## Supplementary Information

**Additional file 1: Fig. S1.** Typical representative images of TEM (**A**–**C**) and HE staining (**D**–**F**) of 3D-CH-IB-ST. **G** Degradation of 3D-CH-IB-ST in rats over 8 weeks. **Fig. S2.** **A**–**D** Representative graphs of phase contrast microscopy (**A**) and immunofluorescence staining (Nestin) (**B**–**D**) of cultured NSCs

## Data Availability

Not applicable.

## Abbreviations

TBI: Traumatic brain injury
HUCMSCs: Human umbilical cord blood mesenchymal stem cells
MTT: Methylthiazolyldiphenyl-tetrazolium bromide
mNSS: Modified neurological severity score
TEM: Transmission electron microscopy
SEM: Scanning electron microscopy
MEP: Motor evoked potential
MWM: Morris water maze
CNS: Central nervous system
MSC: Mesenchymal stem cell
TGFB1: Transforming growth factor beta 1
FGF2: Fibroblast growth factor 2
BDNF: Brain-derived neurotrophic factor
DMEM: Dulbecco’s modified eagle medium
CH: Collagen/heparan sulfate scaffold
3D-CH: 3D-printed collagen/heparan sulfate scaffold
NSCs: Neural stem cells
CNTF: Ciliary neurotrophic factor
NGF: Nerve growth factor
EGF: Epidermal growth factor
GDNF: Glia cell line-derived neurotrophic factor
NT-3: Neurotrophin-3
IGF-1: Insulin-like growth factors-1
VEGF: Vascular endothelial growth factor
bFGF: Beta fibroblast growth factor
PDGF-AA: Platelet-derived growth factor AA
