# Supplementary material for: 3D printing of injury-preconditioned secretome/collagen/heparan sulfate scaffolds for neurological recovery after traumatic brain injury in rats
Source: Stem Cell Res Ther. 2022 Dec 19;13:525. doi: 10.1186/s13287-022-03208-0 (PMC9764714; doi:10.1186/s13287-022-03208-0)
Supplement: Supplementary file 1 — Additional file 1: Fig. S1. Typical representative images of TEM (A–C) and HE staining (D–F) of 3D-CH-IB-ST. G Degradation of 3D-CH-IB-ST in rats over 8 weeks. Fig. S2. A–D Representative graphs of phase contrast microscopy (A) and immunofluorescence staining (Nestin) (B–D) of cultured NSCs [file 13287_2022_3208_MOESM1_ESM.docx]

**Electronic Supplementary Information**

**3D printing of injury-preconditioned secretome/collagen/heparan sulfate scaffolds for neurological recovery after traumatic brain injury in rats**

Xiao-Yin Liu^1,2^*, Zhe-Han Chang^1^*, Chong Chen^1,3^*, Jun-Liang^1^, Jian-Xin Shi^1^, Xiu Fan^1^, Qi Shao^1^, Wei-Wei Meng^1^, Jing-Jing Wang^3^, Xiao-Hong Li^1#^

1. Academy of Medical Engineering and Translational Medicine, Tianjin University, Tianjin 300072, China.

2. Department of Neurosurgery, West China Hospital, West China Medical School, Sichuan University, Chengdu 610041, Sichuan, China.

3. Tianjin Key Laboratory of Neurotrauma Repair, Characteristic Medical Center of People’s Armed Police Forces, Tianjin 300162, China

* Xiao-Yin Liu, Zhe-Han Chang and Chong Chen contributed equally to this study.

^#^Corresponding authors. Tel: +86-022-83612122; Fax: +86-022-83612122; E-mail addresses: [xhli18@tju.edu.cn](mailto:xhli18@tju.edu.cn,)

**Supplementary Data**


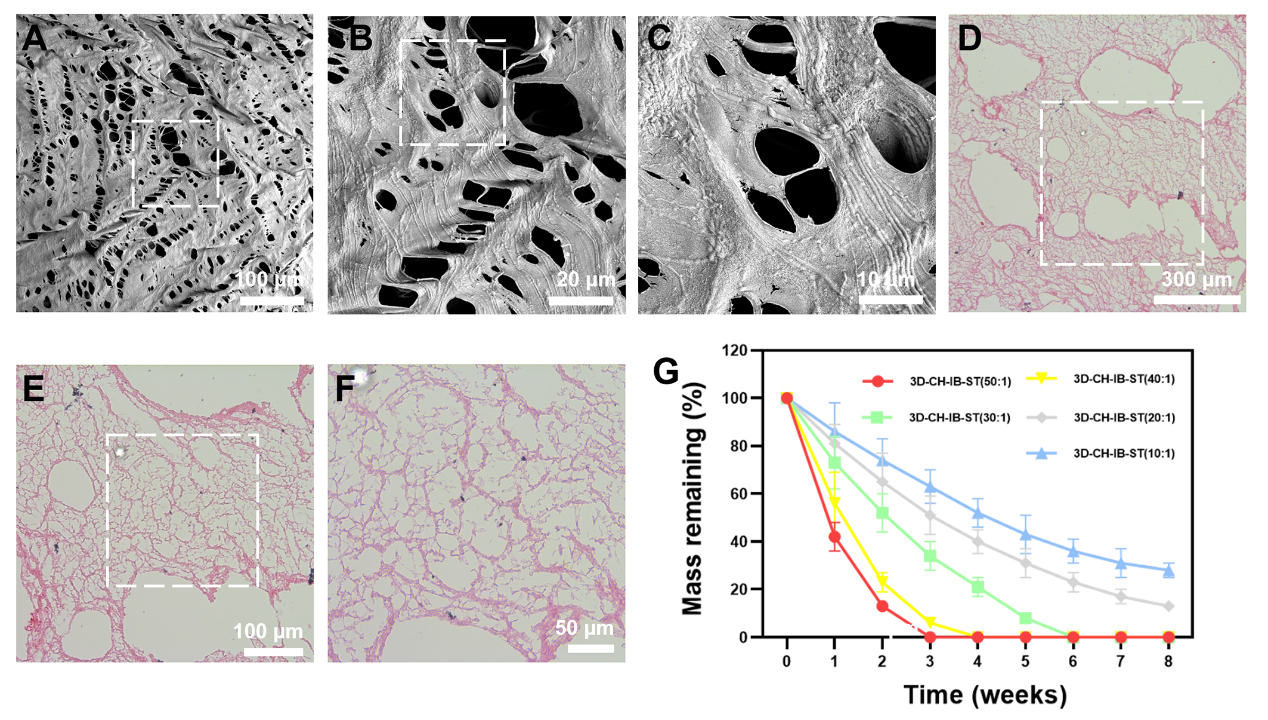


**Fig. S1** Typical representative images of TEM (A-C) and HE staining (D-F) of 3D-CH-IB-ST. (G) Degradation of 3D-CH-IB-ST in rats over 8 weeks.


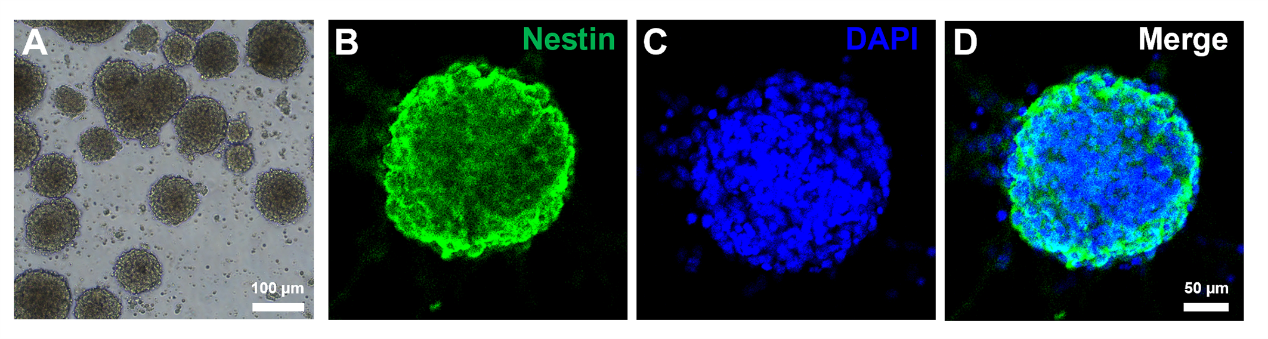


**Fig. S2** (A-D) Representative graphs of phase contrast microscopy (A) and immunofluorescence staining (Nestin) (B-D) of cultured NSCs.
